# CD146 controls the quality of clinical grade mesenchymal stem cells from human dental pulp

**DOI:** 10.1186/s13287-021-02559-4

**Published:** 2021-08-30

**Authors:** Lan Ma, Zhiqing Huang, Di Wu, Xiaoxing Kou, Xueli Mao, Songtao Shi

**Affiliations:** South China Center of Craniofacial Stem Cell Research and Guangdong Province Key Laboratory of Stomatology, Guanghua School and Hospital of Stomatology, Sun Yat-Sen University, Guangzhou, 510055 Guangdong People’s Republic of China

**Keywords:** Human dental pulp stem cells, Quality, Clinical grade, CD146, ERK pathway

## Abstract

**Background:**

Human mesenchymal stem cells from dental pulp (hMSC-DP), including dental pulp stem cells from permanent teeth and exfoliated deciduous teeth, possess unique MSC characteristics such as expression of specific surface molecules and a high proliferation rate. Since hMSC-DP have been applied in numerous clinical studies, it is necessary to establish criteria to evaluate their potency for cell-based therapies.

**Methods:**

We compared stem cell properties of hMSC-DP at passages 5, 10 and 20 under serum (SE) and serum-free (SF) culture conditions. Cell morphology, proliferation capacity, chromosomal stability, surface phenotypic profiles, differentiation and immunoregulation ability were evaluated. In addition, we assessed surface molecule that regulates hMSC-DP proliferation and immunomodulation.

**Results:**

hMSC-DP exhibited a decrease in proliferation rate and differentiation potential, as well as a reduced expression of CD146 when cultured under continuous passage conditions. SF culture conditions failed to alter surface marker expression, chromosome stability or proliferation rate when compared to SE culture. SF-cultured hMSC-DP were able to differentiate into osteogenic, adipogenic and neural cells, and displayed the capacity to regulate immune responses. Notably, the expression level of CD146 showed a positive correlation with proliferation, differentiation, and immunomodulation, suggesting that CD146 can serve as a surface molecule to evaluate the potency of hMSC-DP. Mechanistically, we found that CD146 regulates proliferation and immunomodulation of hMSC-DP through the ERK/p-ERK pathway.

**Conclusion:**

This study indicates that SF-cultured hMSC-DP are appropriate for producing clinical-grade cells. CD146 is a functional surface molecule to assess the potency of hMSC-DP.

**Supplementary Information:**

The online version contains supplementary material available at 10.1186/s13287-021-02559-4.

## Introduction

Mesenchymal stem cells (MSCs) have been used in clinics to treat a variety of human diseases [1–4]. MSCs can be isolated from multiple tissues, such as bone marrow, umbilical cord tissue, adipose tissue and dental pulp [5–7]. The minimal criteria for MSC identification were established by the International Society for Cellular Therapy (ISCT) in 2006 [8]. However, the standards for the quality assessment of MSCs from specific tissue resources have not yet been reported.

Human mesenchymal stem cells from dental pulp (hMSC-DP) have been isolated and extensively studied [9, 10]. Their superior proliferation, multi-differentiation, and immunomodulatory capacities have been reported [11–13]. Recently, hMSC-DP have been applied to regenerate orofacial tissues and treat systemic diseases in pre-clinical and clinical settings [11, 14–18]. It is well-known that the quality and potency of MSCs are critical to ensure their therapeutic effects [19]. However, the minimum criteria for MSCs formulated by ISCT may not provide enough guidance to evaluate the quality and potency of hMSC-DP [8].

Previous studies indicate that fetal bovine serum (FBS)-cultured MSCs may lead to some ethical and immune response issues [20–22]. Obvious quality variation across FBS batches also limits the large-scale manufacture of MSCs. Therefore, it is necessary to use serum-free (SF) culture systems for the future use of MSCs in clinics. FBS substitutes, including human platelet lysate and chemically defined SF media, appear to provide sufficient nutritional support for the culture expansion of hMSC-DP and maintain their regenerative potency [23–25]. However, detailed cultural effects of SF medium on hMSC-DP are not fully understood.

In this study, we established a SF culture system suitable for the evaluation of culture-expanded hMSC-DP. Additionally, we identified that CD146 is a potential functional surface molecule to show the potency of hMSC-DP and established minimal criteria to assess the potency of hMSC-DP.

## Materials and methods

### Mice

Male C57BL/6J mice were purchased from Sun Yat-sen University, Guangzhou, China. All animal experiments were performed under an institutionally approved protocol for the use of animal research (Sun Yat-sen University, SYSU-IACUC-2020-000394).

### Isolation and culture of hMSC-DP

Human exfoliated deciduous incisors and third molars were obtained as discarded biological samples from Guanghua School and Hospital of Stomatology, Sun Yat-sen University. The informed consent forms were acquired from each guardian on behalf of the child donors or from each adult donor, respectively. All teeth were intact without any inflammation. The protocol (KQEC-2020-055-01) was approved by the Medical Ethics Committee of Hospital of Stomatology, Sun Yat-sen University. DPSCs and SHED were isolated and cultured until passage 2 (P2) as reported previously [9, 10]. P2 hMSC-DP were cultured with serum (SE) or serum-free (SF) conditions until P20. SE-hMSC-DP were cultured as previously reported [9, 10]. SF-hMSC-DP were cultured in a high-glucose Dulbecco’s Modified Eagle Medium (DMEM, Invitrogen) supplemented with 5% human platelet lysate (Compass Biomedical), 100 U/ml penicillin, 100 mg/ml streptomycin (Gibco), and 1% GlutaMAX™ Supplement (Gibco). The plastic-adherent confluent cells were passaged with 0.25% trypsin containing 1 mM EDTA (Gibco) and continuously maintained in the SE or SF medium. Approximately 1 × 10^6^ hMSC-DP were selected for inoculation on a 10 cm culture dish (Corning) at each passage, while the remaining cells were stored in serum-free cryopreservation medium (CELLBANKER™ 2) at -80 °C. hMSC-DP at P5, P10 and P20 were used for subsequent tests.

### Antibodies and reagents

Anti-CD34, CD45, CD73, CD90, CD105, CD146 and 7-AAD antibodies were purchased from BD Biosciences. Anti-CD3, CD8 and Annexin V antibodies were purchased from BioLegend. Anti-Runx2, ERK and p-ERK antibodies were purchased from Cell Signaling Technology. Anti-CD146, ALP, βIII-tubulin and NeuN antibodies were purchased from Abcam. Anti-LPL and β-actin antibodies were purchased from Invitrogen. Anti-PPARγ antibody was purchased from Santa Cruz. WGA-488 was purchased from Biosharp. Hoechst 33,342 was purchased from Sigma. The kFluor488-EDU cell proliferation assay kit was purchased from KeyGEN Biotech. Lipofectamine™ RNAiMAX transfection reagent was purchased from Invitrogen. CD146 siRNA was purchased from RIBO Biotech. PD98059 was purchased from Beyotime. Cellular Senescence Assay Kit was purchased from Merck Millipore. Dextran Sulfate Sodium (DSS) was purchased from MP Biochemicals.

### Flow cytometry analysis

For cell surface marker analysis, hMSC-DP suspension from P5, P10 and P20 were prepared at a density of 10^6^ cells per 100 μl. Thereafter, 1 μl antibody was added to 100 μl cell suspension and incubated for 30 min at 4 °C in the dark, followed by analysis using flow cytometry (ACEA NovoCyte™). The apoptotic T cells were stained with CD3 and 7AAD antibodies at 4 °C in the dark for 30 min and washed twice with PBS, followed by incubation with Annexin V antibody.

### In vitro immunomodulatory capacity of hMSC-DP

hMSC-DP at P5, P10 and P20 (1 × 10^5^/per well) were seeded on a 12-well culture plate (Corning) and incubated for 24 h. T cells, pre-stimulated by anti-CD3 and CD8 antibodies, were directly loaded onto hMSC-DP and cocultured for 2 days. The number of apoptotic T cells was detected by flow cytometry.

### Karyotype analysis

Karyotype analysis of hMSC-DP, including chromosome numbers and G-banded karyotypes, was performed by using a kit from HaoGe Biotechnology Corporation Limited (Shanghai, China).

### EdU incorporation assay

hMSC-DP (2 × 10^4^ cells per well) were seeded in 24-well plates (Corning) and incubated at 37 °C for 24 h. Cells were treated with 5-ethynyl-20-deoxyuridine (EdU) at a working concentration of 50 μM in a 200 μl culture medium for 2 h. Then cells were detected using an EdU cell proliferation assay kit (KeyGEN BioTECH) according to the manufacturer’s instructions.

### Population doublings

hMSC-DP at P4 were seeded at 2 × 10^5^ cells in 60 mm dishes (Corning) in SE or SF culture medium. Cells were harvested and seeded at the same number when they reached around 70–80% confluence. Population doublings (PD) were calculated by the following formula: PD = log2 (number of harvested cells/number of seeded cells). PD numbers were determined by the cumulative addition of total numbers generated from P5 to the end of cell division. The PD assay was repeated with 3 independent isolated cells for each group.

### Colony-forming unit (CFU) assay

A total number of 1000 hMSC-DP from each group were cultured and plated in 6 cm dishes (Corning). After 10 days, cells were fixed with 4% paraformaldehyde and stained with 1% crystal violet for 5 min. Colonies containing 50 or more cells were selected. The experiment was repeated three times.

### Cell morphology analysis by high-content imaging

3000 hMSC-DP of each group were seeded in 96-cell well plates (Corning) and incubated for 24 h. Adherent cells were washed with PBS and then fixed in 4% formaldehyde for 10 min at room temperature. Cells were stained with Phalloidin, WGA, and Hoechst 33,342. Plates were scanned in the Operetta CLS and cells of each group were analyzed for cell size and shape using the Harmony software (PerkinElmer).

### Cellular senescence assay

A total number of 5 × 10^4^ hMSC-DP from each group were seeded in 24-well plates (Corning) and incubated for 24 h. Then a cellular senescence assay was conducted following the protocol provided with the Cellular Senescence Assay Kit (Merck Millipore).

### Induction of osteogenic, adipogenic and neurogenic differentiation

Approximately 3 × 10^5^ hMSC-DP of each group were cultured in 6 cm plates until they reached full confluence. The osteogenic and adipogenic differentiation induction and assessment were conducted as reported previously [26, 27]. For neurogenic differentiation, a total of 4 × 10^4^ cells was seeded on a 24-well culture plate (Corning) and incubated for 24 h. Then cells were changed to neural differentiation medium and cultured for 10 days. The neural differentiation assessment was conducted as reported previously [10].

### siRNA transfection and chemical treatment

For siRNA transfection, hMSC-DP (5 × 10^5^/well) were seeded in a 6-well culture plate in a low-serum medium (Opti-MEM, Gibco) and treated with CD146 siRNA (RIBO, China) or control vehicle siRNA with lipofectamine reagent (Invitrogen) for 24 h, according to the manufacturer’s instructions. For chemical reagent treatments, hMSC-DP were treated with 10 μM PD98059. Treated cells were harvested or used for further experiments.

### Allogenic hMSC-DP transplantation in acute colitis mice

Acute colitis was induced by administering 3% DSS (MP Biochemicals) dissolved in drinking water for 8 days. A total number of 1 × 10^6^ hMSC-DP from each group were infused into colitis mice intravenously at day 3 after feeding with DSS water. All mice were euthanized and analyzed on day 8. Induced colitis mice were evaluated as previously described [28].

### Statistical analysis

GraphPad Prism 8 was used to perform statistical analysis. Comparisons between two groups were analyzed using independent two-tailed Student’s *t* tests, while comparisons between more than two groups were analyzed using one-way ANOVA. *P* values < 0.05 were considered statistically significant.

## Results

### Morphological characteristics of hMSC-DP

**Fig. 1:**
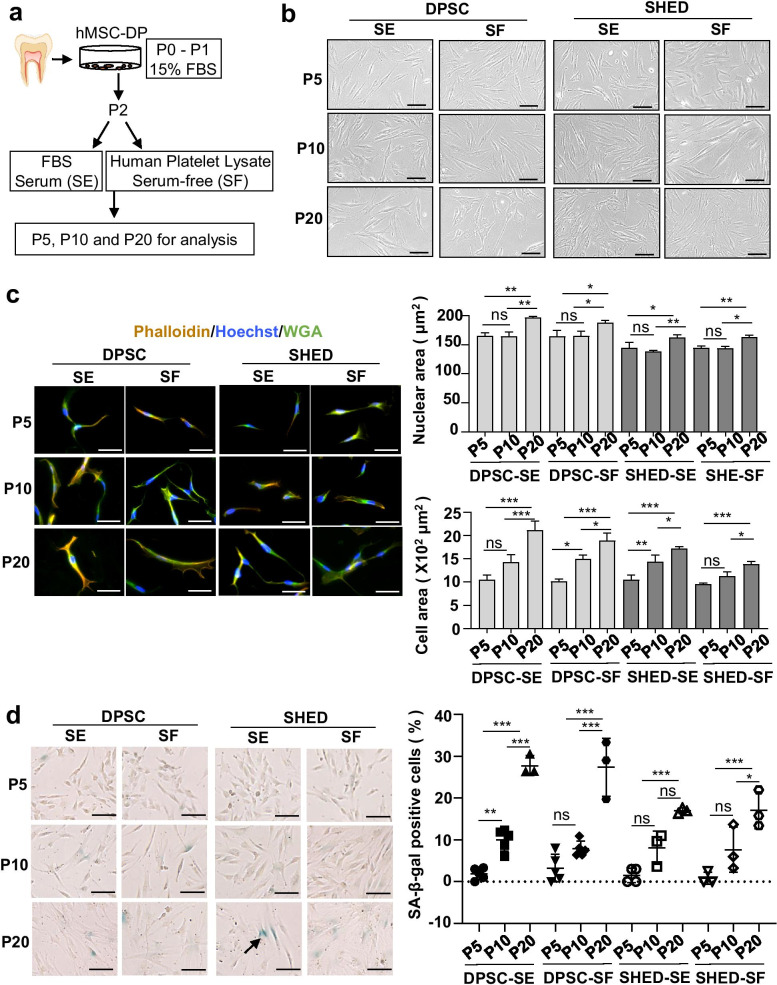
Morphological characteristics of hMSC-DP with accumulated passages. **a** Schema of isolation and culture of hMSC-DP under SE or SF culture conditions. **b** The morphology of P5, P10 and P20 hMSC-DP under SE or SF culture conditions. Scale bar = 50 μm. **c** Cell morphology analysis by high-content imaging. hMSC-DP were co-stained with Phalloidin (orange), Hoechst (blue) and WGA (green). Scale bar = 100 μm. *n* = 3 for each group. **d** Cellular senescence assay. The percentage of SA-β-gal positive cells was calculated and compared across groups at P5, P10 and P20. The black arrow indicates positive staining of SA-β-gal. Scale bar = 100 μm. *n* = 3 ~ 5 for each group. SE, serum; SF, serum-free. Data shown as mean ± SEM. *p < 0.05, **p < 0.01, ***p < 0.001. *ns* not significant

We isolated DPSCs from the third molars of three adult donors and SHED from deciduous incisors of three 6–8 years old donors. All donors were healthy volunteers and the teeth were devoid of caries and inflammation. hMSC-DP, including DPSCs and SHED, were isolated from dental pulp according to the previous reports [9, 10]. hMSC-DP at P2 were separated to be propagated under traditional FBS (SE) or human platelet lysate serum-free (SF) culture conditions to generate DPSC-SE, DPSC-SF, SHED-SE and SHED-SF (Fig. 1a).

Cell morphology is regarded as an MSC potency indicator [29]. hMSC-DP from all donors under SE and SF culture conditions adhered to culture dishes and showed elongated and spindle-shaped morphology at P5 and P10. With continuous passage, hMSC-DP became enlarged, cuboidal, and flattened at P20 (Fig. 1b). To further analyze cellular morphological characteristics, we used a high-content imaging approach to show Hoechst-positive nuclei, Phalloidin-positive cytoskeleton and wheat germ agglutinin (WGA)-positive cell membrane [30]. More than 500 cells of each group were analyzed. The nuclear size of hMSC-DP at P20 was significantly larger than P5 in the DPSC-SE (*P* < 0.01), DPSC-SF (*P* < 0.05), SHED-SE (*P* < 0.05) and SHED-SF (*P* < 0.01) groups. The cell size of hMSC-DP at P20 also showed a tendency to become larger when compared to hMSC-DP at P10 (Fig. 1c). The nuclear size showed no significant difference between SE and SF culture conditions (Additional file 1: Fig. S1a), but the cell size of SHED tended to be smaller in SF culture compared to SE culture (*P* < 0.05 at P10, *P* < 0.01 at P20) (Additional file 1: Fig. S1b). Since cell senescence may be associated with alteration of cellular and nuclear size [31], we assessed β-galactosidase (β-gal)-positive senescent hMSC-DP at P5, P10 and P20. We showed that the percentage of β-gal positive cells increased from P5 to P20 in each group, which was statistically significant when compared between P5 hMSC-DP and P20 hMSC-DP (*P* < 0.001 in each group) (Fig. 1d). However, there was no statistically significant difference between SE and SF culture conditions (Additional file 1: Fig. S1c). These data indicate that continuous passage affects hMSC-DP morphological characteristics.

### Proliferation capacity and chromosomal stability of hMSC-DP

**Fig. 2:**
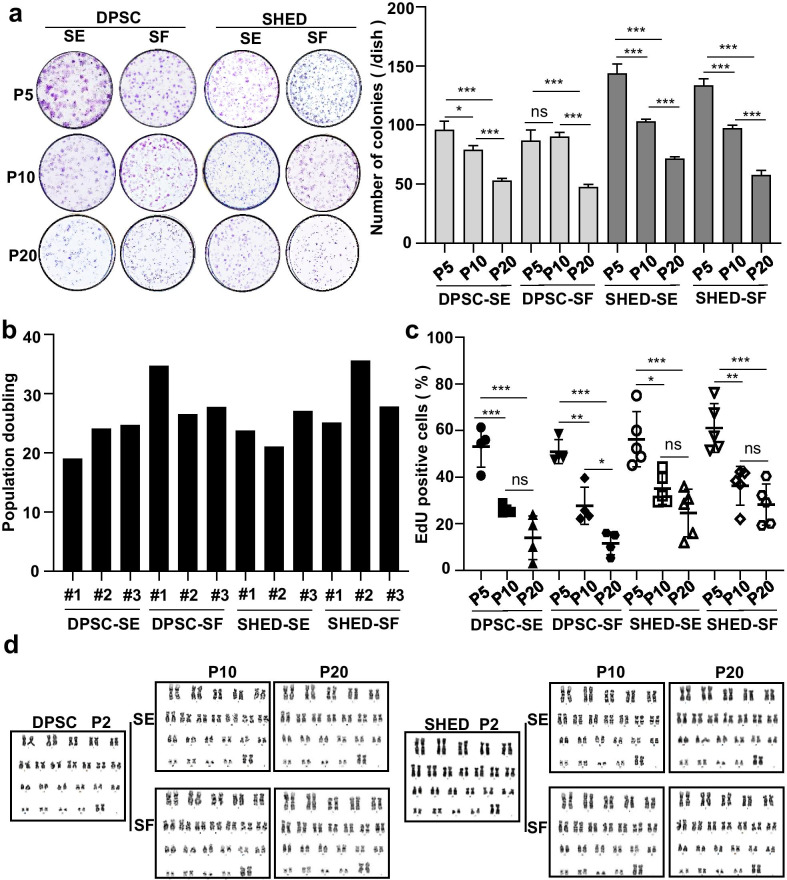
Proliferation capacity and chromosomal stability of hMSC-DP at different passages. **a** Colony-forming ability of DPSCs and SHED under SE and SF culture conditions was assessed by CFU-F assays. The numbers of colonies at P5, P10 and P20 were calculated and compared. *n* = 3 for each group. **b** Proliferation capacity of three groups of donor-derived DPSCs and three groups of donor-derived SHED was analyzed by population doubling assay. **c** Proliferation rate of hMSC-DP was assessed by EdU staining. The percentage of EdU-positive cells was calculated at P5, P10 and P20. **d** Chromosomal stability was assessed by karyotype analysis. The karyotypes of DPSCs and SHED at P5, P10 and P20 were examined. SE, serum; SF, serum-free. Data shown as mean ± SEM. *p < 0.05, **p < 0.01, ***p < 0.001. *ns* not significant

Proliferation capacity is an important parameter for evaluating the potency of MSCs. hMSC-DP were seeded at a density of 1,000 cells/plate and cultured for 10 days. The number of colonies was significantly decreased from P5 to P20 (Fig. 2a). Interestingly, the SHED-SE group showed a higher number of colony formation compared to the SHED-SF group at P20 (Additional file 1: Fig. S2a). To examine the proliferation potential of hMSC-DP, population doubling scores were calculated from three donor-derived batches of DPSCs and three donor-derived batches of SHED. These cells showed similar proliferation capacities (Fig. 2b) and had no significant differences under either SE or SF culture conditions (Additional file 1: Fig. S2b). Also, 5-Ethynyl-2′-deoxyuridine (EdU) staining showed the proliferation rate of hMSC-DP decreased with the continuous passage, and significantly reduced at P20 compared to P5 (Fig. 2c) (*P* < 0.001 at each group). SE and SF culture showed no differences in terms of EdU incorporation (Additional file 1: Fig. S2c).

It is critical to use clinical-grade human MSCs with normal genetic karyotypes and chromosomal stability [32]. We assessed the karyotypes of DPSCs and SHED at P5, P10 and P20 under SE and SF culture conditions. There was no detectable chromosomal aberration in any of the tested groups (Fig. 2d), consistent with the previous reports that human MSCs maintain relatively stable genomes during culture expansion [33, 34].

### Surface phenotypic profiles and in vitro immunoregulation ability of hMSC-DP

**Fig. 3:**
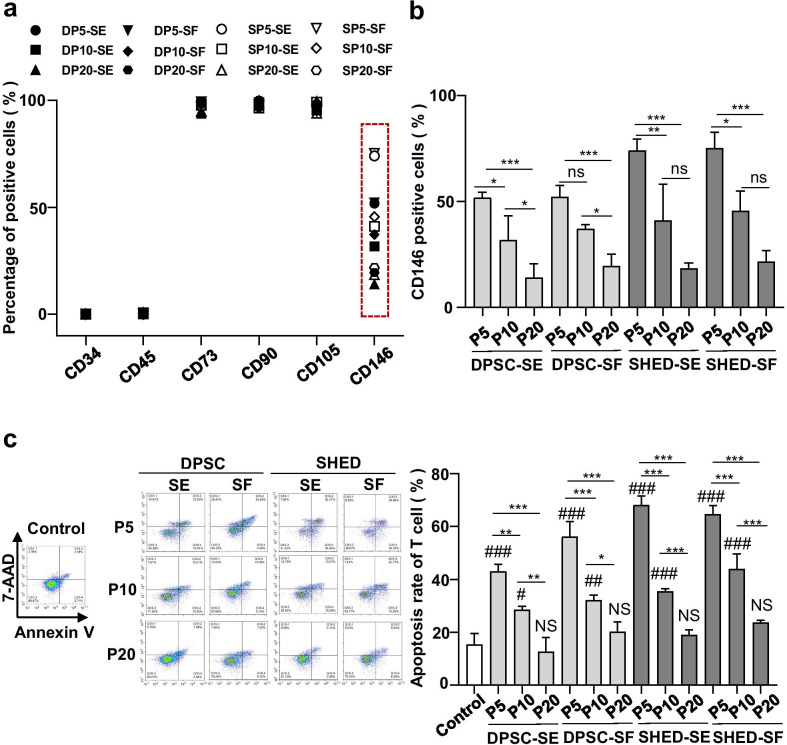
Surface phenotypic profiles and in vitro immunoregulation ability of hMSC-DP at different passages. **a** MSC surface marker analysis of hMSC-DP at P5, P10 and P20 under SE and SF culture conditions was assessed by flow cytometry. The mean positive rates of CD34, CD45, CD73, CD90, CD105 and CD146 are shown. *n* = 3 for each group. **b** The percentage of CD146 positive cells was calculated at P5, P10 and P20 in DPSCs and SHED, independently. *n* = 3 for each group. **c** To assess the in vitro immunoregulation ability, hMSC-DP at P5, P10 and P20 were co-cultured with T cells and the percentage of apoptotic T cells was examined by flow cytometry. *n* = 3 for each group. Data shown as mean **(a)** or mean ± SEM (**b**, **c**). Comparisons with control group: ^#^*p* < 0.05, ^##^*p* < 0.01, ^###^*p* < 0.001. *NS* no significant difference from control group. Comparisons among hMSC-DP at P5, P10 and P20: *p < 0.05, **p < 0.01, ***p < 0.001. *ns* not significant

We next analyzed a series of surface markers including those described by the ISCT [8]. hMSC-DP at P5, P10 and P20 under both SE and SF culture conditions expressed MSC surface markers CD73, CD90 and CD105 while they did not express hematopoietic markers CD34 and CD45 (Fig. 3a). Notably, the expression level of CD146 exhibited heterogeneity across groups (Fig. 3a, Additional file 1: Fig. S3a). We observed a gradually decreased expression of CD146 from P5 to P10 as well as from P10 to P20. There was a significant reduction of CD146 expression at P20 when compared to P5 (DPSC-SE, DPSC-SF, SHED-SE and SHED-SF: *P* < 0.001) (Fig. 3b). Neither SE nor SF culture conditions showed a regulatory effect on the expression of CD146 (Additional file 1: Fig. S3b). Previous studies showed that CD146 is not only a melanoma cell adhesion molecule but also a cellular surface receptor of various ligands, participating in numerous physiological and pathological processes [35]. Importantly, CD146 has been identified as an MSC surface marker [36].

To assess the in vitro immunomodulatory capacity of hMSC-DP, we co-cultured hMSC-DP with activated T cells for 2 days. Flow cytometry analysis showed that hMSC-DP at P5 and P10 were capable of inducing a significant number of T cell apoptosis compared to the control group, but hMSC-DP at P20 lost their capacity to induce T cell apoptosis (Fig. 3c). hMSC-DP under SF culture condition showed a tendency to possess elevated immunomodulatory capability compared to SE-cultured hMSC-DP, though only DPSCs at P5 under SF culture conditions showed significantly elevated immune regulation capacity compared to SE-cultured DPSCs (*P* < 0.05) (Additional file 1: Fig. S3c). These results indicate that hMSC-DP cultured in SF media may be more suitable for using in immunotherapy.

### Multilineage differentiation of hMSC-DP

**Fig. 4:**
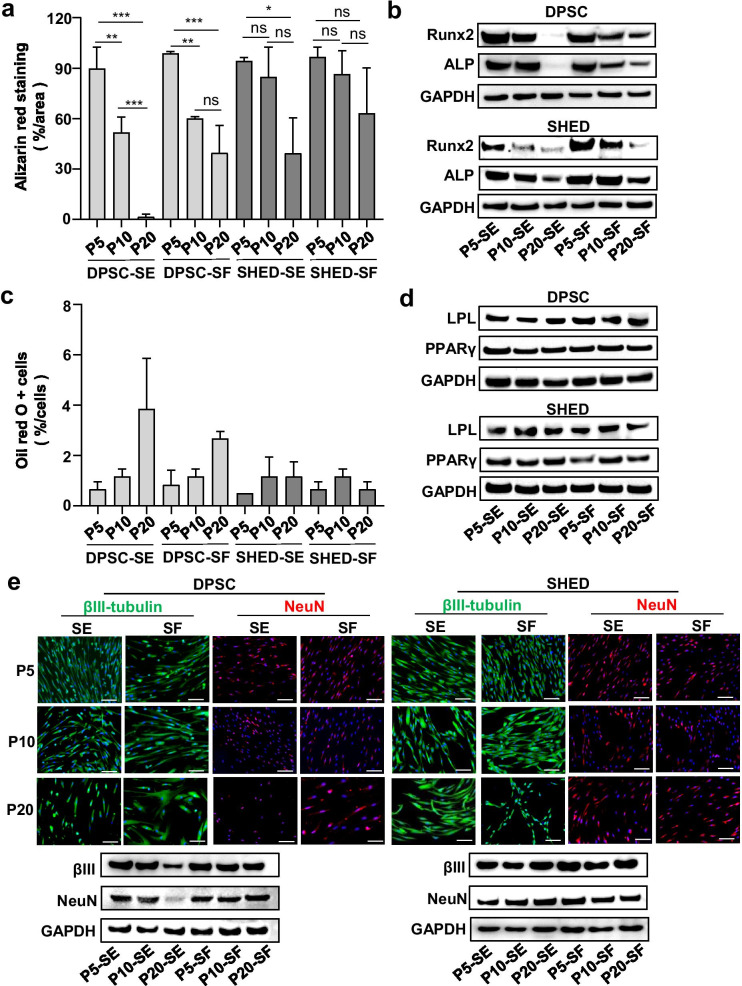
Multilineage differentiation of hMSC-DP. **a**, **b** Osteogenic capacity of hMSC-DP. The percentage of alizarin red staining compared at P5, P10 and P20 (**a**). Western blot analysis showed the expression levels of osteogenic markers including Runx2 and ALP (**b**). *n* = 3 for each group. **c**, **d** Adipogenic capacity of hMSC-DP. The percentage of oil red o positive cells was calculated and analyzed at P5, P10 and P20 (**c**). Western blot analysis showed the expression levels of adipogenic markers including LPL and PPARγ (**d**). *n* = 3 for each group. **e** Neural differentiation of hMSC-DP. Immunofluorescent staining and western blot analysis showed the expression of neural markers βIII-tubulin and NeuN. Scale bar = 100 μm. SE, serum; SF, serum-free. Data shown as mean ± SEM. *p < 0.05, **p < 0.01, ***p < 0.001. *ns* not significant

Multilineage differentiation potential is a critical parameter in evaluating the potency of MSCs. To assess the osteogenic capacity of hMSC-DP, we cultured hMSC-DP at P5, P10 and P20 in SE and SF osteogenic inductive media for two weeks. Alizarin red staining showed that hMSC-DP from P5 to P20 showed a tendency towards reduced mineralized nodule formation, indicating that continued passage impairs the osteogenic differentiation potential of hMSC-DP (Fig. 4a, Additional file 1: Fig. S4a). Western blot analysis confirmed that the continuous passage reduced the expression levels of runt-related transcription factor 2 (Runx2) and alkaline phosphatase (ALP) (Fig. 4b). Interestingly, SHED at P20 under SF culture conditions still possessed relatively strong osteogenic capacity, while DPSCs at P20 under SF culture conditions had a significantly elevated capacity to form mineralized nodules when compared to SE-cultured DPSCs (Additional file 1: Fig. S4a). All these results suggest that SF medium has advantages in maintaining the osteogenic capacity of hMSC-DP.

Our previous studies showed that hMSC-DP are capable of differentiating into adipocytes, but have a limited capacity to do so [9, 10]. Here, we showed that hMSC-DP from P5 to P20 under SE or SF culture medium exhibited limited adipogenic differentiation capacity after 6 weeks of adipogenic induction (Fig. 4c, Additional file 1: Fig. S4b). Western blot analysis showed the expression levels of two adipocyte-specific transcripts, peroxisome proliferator-activated receptor-γ2 (PPARγ) and lipoprotein lipase (LPL), in each group (Fig. 4d).

Previous studies showed that DPSCs and SHED are capable of differentiating into neural cells, presumably due to their neural crest origin [37–39]. Here, we showed that hMSC-DP from P5 to P20 under SE or SF culture conditions maintain neurogenic differentiation capacity, as assessed by western blot and immunostaining to show expression of neuronal markers βIII tubulin and NeuN when cultured under neurogenic inductive conditions (Fig. 4e).

### hMSC-DP ameliorate Dextran sulfate sodium (DSS)-induced experimental colitis

DSS-induced experimental colitis has been widely used to evaluate the in vivo immunomodulatory capacity of MSCs [28, 40]. To further assess the immunomodulatory ability of hMSC-DP, we systemically infused SE and SF-cultured hMSC-DP at P5, P10 and P20 into experimental colitis mice on day 3 after 3% DSS induction. The experimental colitis mice were sacrificed at 8 days post DSS induction. On day 8, DPSC-SE at P5 and all DPSCs cultured under SF conditions improved the body weight of experimental colitis mice after intravenous infusion. DPSC-SF at P5 showed a superior capacity to rescue the body weight when compared to DPSC-SF at P10 (*P* < 0.05) and P20 (*P* < 0.01).

**Fig. 5:**
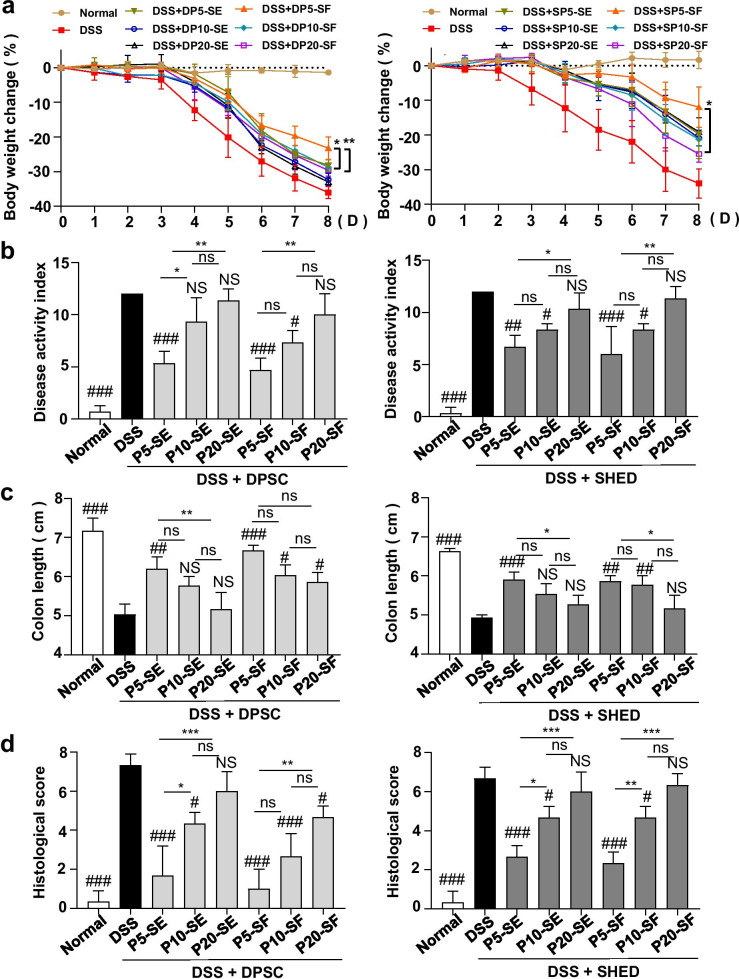
hMSC-DP ameliorate Dextran sulfate sodium (DSS)-induced experimental colitis. **a** Body weights were recorded every day for 8 days in DPSCs and SHED transplanted groups. The relative alteration of body weight was calculated. *n* = 3 for each group. **b** Disease activity index (DAI) was evaluated on day 8 of DSS feeding. *n* = 3 for each group. **c** Colon length was measured on day 8. *n* = 3 for each group. **d** Histological score based on H&E staining images was assessed on day 8. The histological score of each group was calculated. *n* = 3 for each group. SE, serum; SF, serum-free. Data shown as mean ± SEM. Comparisons with untreated DSS group: ^#^*p* < 0.05, ^##^*p* < 0.01, ^###^*p* < 0.001. *NS* not significantly different from DSS group. Comparisons of DPSCs or SHED at P5, P10 and P20: *p < 0.05, **p < 0.01, ***p < 0.001. *ns* not significant

Intravenously infused SHED, except SHED-SF at P20, significantly elevated the body weight in DSS-induced colitis mice when compared to the DSS group. SHED-SF at P20 somewhat lost the ability to improve the body weight (*P* < 0.05) (Fig. 5a). Treatment with hMSC-DP at P5 resulted in a significantly improved disease activity index (DAI) compared to the untreated DSS group, whereas hMSC-DP at P20 failed to improve the DAI (Fig. 5b). Anatomical images of the colon of each group showed that DPSC-SE at P5 and all DPSCs under SF culture conditions had therapeutic effects for DSS-induced colitis, while SHED-SE at P5 and SHED-SF at P5 and P10 improved the colon length compared to the untreated DSS group (Fig. 5c, Additional file 1: Fig. S5b). Histological scores from HE staining images showed that hMSC-DP from all groups, except DPSC-SE at P20 and SHED at P20, offered effective therapy for DSS-induced colitis (Fig. 5d, Additional file 1: Fig. S5c). In general, the immunoregulatory ability of stem cells decreased gradually with the continuous passage. The therapeutic effect of P10 hMSC-DP was weaker than that of P5 hMSC-DP in experimental colitis mice, while P20 hMSC-DP showed little therapeutic effect.

In general, culture conditions have little effect on the immunomodulatory capacity of SHED. However, SF-cultured DPSCs tend to exhibit a greater immunomodulatory effect than SE-cultured DPSCs, but the difference is not statistically significant (Additional file 1: Fig. S5).

### CD146 regulates the potency of hMSC-DP

**Fig. 6:**
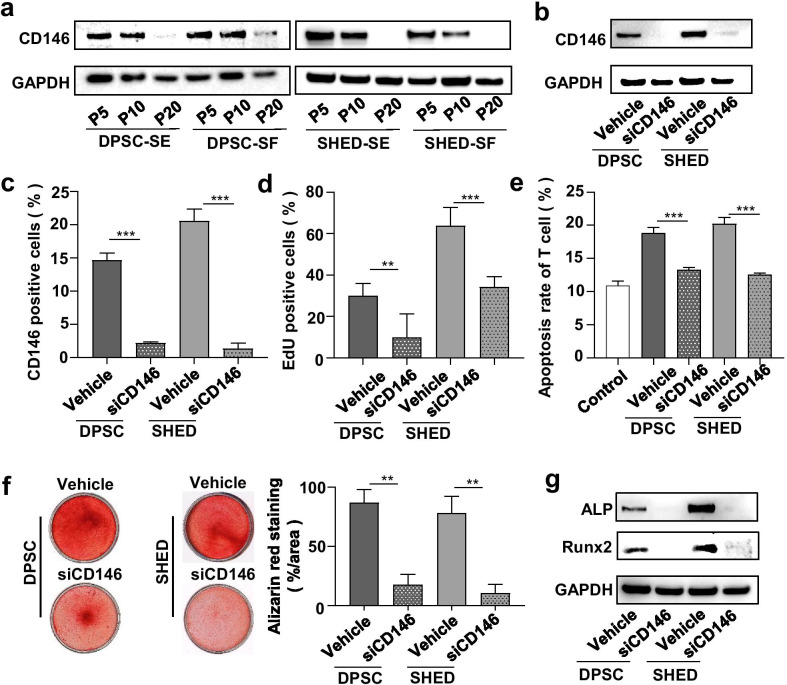
CD146 expression is associated with hMSC-DP proliferation, differentiation and immune modulation. **a** Western blot analysis showed the expression of CD146 in DPSCs and SHED at P5, P10 and P20 under SE or SF culture conditions. **b** The expression level of CD146 in DPSCs and SHED at P10 was downregulated by siCD146, as assessed by western blot. **c** Flow cytometry analysis showed the percentage of CD146-positive cells was reduced after siCD146 treatment. *n* = 3 for each group. **d** EdU staining showed the proliferation rate of hMSC-DP was decreased after siCD146 treatment. *n* = 3 for each group. **e** After co-culturing siCD146-transfected hMSC-DP with T cells, the percentage of apoptotic T cells was reduced, as assessed by flow cytometry. *n* = 3 for each group. **f**, **g** Osteogenesis of hMSC-DP was impaired by knocking down CD146, as assessed by the percentage of alizarin red staining (**f**) and western blot of osteogenic markers (**g**). *n* = 3 for each group. SE, serum; SF, serum-free. Data shown as mean ± SEM. **p < 0.01, ***p < 0.001. *ns* not significant

Previous studies showed that MSCs with high CD146 expression have superior osteogenic and immunoregulatory capacity relative to those with low CD146 expression [41–43]. We found that the expression level of CD146 in hMSC-DP decreased with passage (Fig. 6a). To further explore the role of CD146 in hMSC-DP, we blocked the expression of CD146 in hMSC-DP with siCD146 (Fig. 6b, c), as shown by western blot and flow cytometry. After siCD146 interference, the capacities for proliferation, in vitro immunoregulation and osteogenesis were significantly decreased, as indicated by EdU staining, apoptosis rate of T cells, alizarin red staining and western blot analysis of ALP and Runx2 (Fig. 6d–g). Furthermore, we analyzed the correlation between the potency of hMSC-DP and the expression level of CD146 and found that the potency of hMSC-DP is positively correlated with the expression level of CD146, especially in proliferation, osteogenesis, and in vivo immunoregulatory capacity (Additional file 1: Fig. S6). These results suggest that CD146 is a functional surface molecule that represents the potency of hMSC-DP.

**Fig. 7:**
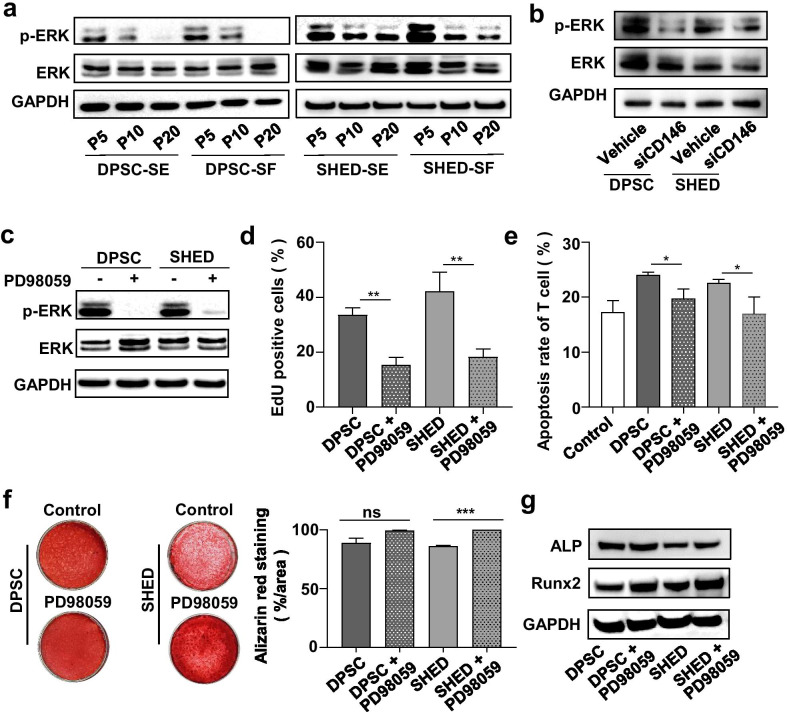
CD146 controls hMSC-DP proliferation and immune modulation through ERK pathway. **a** Western blot analysis showed the expression level of ERK pathway in DPSCs and SHED at P5, P10 and P20 under SE or SF culture conditions. **b** ERK pathway was downregulated by siCD146 treatment in hMSC-DP, as shown by western blot analysis. **c** The expression level of p-ERK was inhibited by ERK pathway inhibitor PD98059. **d** EdU staining showed the proliferation rate of hMSC-DP was reduced after treatment with PD98059. *n* = 3 for each group. **e** After co-culturing PD98059 treated hMSC-DP with T cells, the percentage of apoptotic T cells was decreased, as shown by flow cytometry. *n* = 3 for each group. **f**, **g** Osteogenesis of hMSC-DP was examined after PD98059 treatment, as assessed by the percentage of alizarin red staining (**f**) and western blot analysis of osteogenic markers (**g**). *n* = 3 for each group. SE, serum; SF, serum-free. Data shown as mean ± SEM. *p < 0.05, **p < 0.01, ***p < 0.001. *ns* not significant

It is well-known that ERK/p-ERK pathway plays a critical role in regulating MSC proliferation and differentiation [27, 44, 45]. Here, we showed that the expression level of p-ERK, but not ERK, was consistent with the expression level of CD146 in hMSC-DP (Fig. 7a). Western blot results showed that siCD146 treatment downregulated the expression of p-ERK in hMSC-DP (Fig. 7b), suggesting that CD146 may maintain the potency of hMSC-DP through ERK/p-ERK pathway. To test this hypothesis, we used PD98059, an ERK inhibitor, to reduce the expression of pERK in hMSC-DP, as assessed by western blot (Fig. 7c). ERK inhibitor treatment significantly reduced the proliferation rate and immunosuppression capacity of hMSC-DP, as assessed by EdU staining and T cell coculture assay (Fig. 7d, e). Interestingly, osteogenic differentiation was not affected in DPSCs group but elevated in SHED group after treated with a p-ERK inhibitor, as reported previously [27, 46]. These results indicate that CD146 may maintain hMSC-DP proliferation and immune modulation capacities, but not osteogenic differentiation capacity, via ERK pathway.

## Discussion

SHED and DPSCs possess elevated proliferation, differentiation and immunoregulation capacities [12, 13]. Recently, hMSC-DP have been used in clinics for tissue regeneration and immune therapies (Additional file 2: Table S1). Therefore, it is necessary to develop standard operation procedures (SOPs) for the clinical use of hMSC-DP. These SOPs should ultimately provide guidance and acceptance criteria for all necessary steps, such as clinical-grade cell preparation, transportation, biosafety tests, quality control and therapeutic methods [15]. Among these criteria, the quality and potency of hMSC-DP are critical for their therapeutic effects.

FBS has been generally used as an essential component for MSC culture [47–49]. However, it is becoming increasingly common to use serum-free culture to expand MSCs for clinical application [25, 47, 48]. Human platelet lysate shows benefits in promoting MSC proliferation and thus may serve as a possible alternative to replace FBS [47]. Here, we used human platelet lysate-based serum-free medium to culture hMSC-DP and found that this culture medium is appropriate for expansion of hMSC-DP in terms of achieving normal morphology, cell proliferation, multipotency, immunoregulation, surface molecule expression, and karyotypes.

MSCs can be derived from multiple tissues, but MSCs from different tissue sources may possess unique characteristics [49]. hMSC-DP are derived from neural crest cells and easily differentiate into neural cells [37]. Here, we further reveal that hMSC-DP maintain neural differentiation potential even at passage 20. Additionally, we show that hMSC-DP have osteogenic differentiation potential and maintain their osteogenic capacity even after continuous passage in SF culture conditions. As dental pulp-derived MSCs, hMSC-DP possess unique characteristics including elevated proliferation and the potential for neural and odonto/osteo-differentiation [9, 10, 50]. Accurate evaluation of hMSC-DP characteristics can help to ensure their quality for future clinical application.

MSC-mediated immunomodulation and immune therapies have been widely reported [51–53]. The MSC Committee of the ISCT published a working proposal for the immunological characterization of MSCs in 2013, including MSC immunophenotyping, an in vitro cell responder assay and in vivo animal models [54]. In this study, we found that hMSC-DP at early passages such as P5 show optimal immunoregulation effects in vitro in a T cell coculture system and in vivo in a DSS-induced colitis mouse model. However, continued passaging (to P10 and beyond) reduces their therapeutic capacity for colitis mice, which may relate to their diminished ability to induce T cell apoptosis [28].

CD146 was initially identified as a specific marker of malignant melanoma [55]. Previous studies showed that CD146 is expressed on the surface of human bone marrow MSCs, human umbilical cord-derived MSCs, human adipose tissue-derived MSCs, DPSCs and SHED [36, 42, 56–58]. MSCs with high expression of CD146 show elevated osteogenic and immunoregulatory abilities compared to those with low CD146 expression [41, 42, 56]. Here, we found that the expression levels of traditional MSC surface markers, including CD73, CD90 and CD105, fail to reflect the potency of hMSC-DP, while the expression level of CD146 correlates with hMSC-DP capacity for proliferation, differentiation, and immunoregulation [26, 59]. Therefore, we propose CD146 as a functional surface molecule to predict the quality of hMSC-DP.

ERK/p-ERK pathway plays a critical role in regulating proliferation, immunoregulation and differentiation of MSCs [27, 60, 61]. Previous studies showed that CD146 expression is associated with the activation of ERK pathway during epithelial-mesenchymal transition [62] and tumor angiogenesis [63]. In this study, we demonstrated that CD146 maintained cell proliferation and immunomodulation through ERK/p-ERK pathway, but not osteogenic differentiation. Usually, a highly proliferative state is incompatible with differentiation in MSCs. The controversial role of ERK signaling has been discussed in the context of osteogenic differentiation of MSCs [45]. Activation of ERK signaling in human bone marrow-derived MSCs promotes osteogenic differentiation [64, 65], while upregulation of ERK/p-ERK pathway contributes to the suppression of osteogenesis of MSCs [27, 44, 46]. ERK/p-ERK may regulate other pathways, such as PI3-kinase/Akt or P38 pathway, to affect osteogenic differentiation of hMSC-DP [66, 67].

The minimal criteria for defining MSCs proposed by ISCT are quite basic and general [8], but it may fail to totally reflect the comprehensive characteristics of MSCs, such as their trophic activity [68] and immunomodulatory capacity [28]. Therefore, it may be insufficient to predict the potency of MSCs for clinical applications. Moreover, different tissue-derived MSCs may exhibit parent tissue specificity and possess different functional potential [69]. With the improvement of our understanding of the functional and tissue-specific characteristics of MSCs, it is necessary to define the criteria of tissue-specific MSCs for translational precision therapies. To meet upcoming requirements for defining optimal MSCs for clinical application, we propose additional criteria to define the potency of hMSC-DP: (1) Adherence to plastic forming CFU-F in serum-free culture conditions; (2) CD146 expressed by over 30% of cells; (3) Neural differentiation potential. These added criteria aim for standardized identification of hMSC-DP for clinical use. In our study, we found that 10–80% of SHED and 5–70% of DPSCs expressed CD146, which was positively correlated with stem cell function. Some critical capacities of hMSC-DP were significantly decreased with the reduced expression of CD146 at passage 10. The positive rate of CD146 expression detected by flow cytometry was about 30–40% at passage 10 in our study. Therefore, we propose that CD146 can serve as a functional surface molecule to evaluate the potency of hMSC-DP. When CD146 positive rate is above 30%, hMSC-DP can provide optimal therapeutic effect in DDS-induced colitis mouse model.

## Conclusion

We explored the physiological and functional status of hMSC-DP in the SF culture system and established minimal criteria to identify the potency of hMSC-DP for potential clinical application. CD146 is a functional surface molecule that reflects the potency of hMSC-DP.

## Supplementary Information

**Additional file 1: Fig. S1** Characterization of the morphology of hMSC-DP under SF culture conditions. **a-b** Cell morphology analysis by high-content imaging. The nuclear area **(a)** and cell area **(b)** of DPSCs and SHED are shown in SE and SF culture conditions. n = 3 for each group. **c** Cellular senescence assay. The percentage of SA-β-gal positive cells was calculated and compared between SE and SF culture conditions. n = 3 ~ 5 for each group. SE, serum; SF, serum-free. Data shown as mean ± SEM. ns, not significant. **Fig. S2** Proliferation capacity of hMSC-DP under SF culture conditions. **a** CFU-F assay. The numbers of colonies of hMSC-DP under SE or SF culture conditions were calculated and compared. n = 3 for each group. **b** Population doubling scores were calculated and compared between SE and SF culture conditions in DPSCs and SHED groups, independently. n = 3 for each group. **c** EdU assay. The percentage of EdU-positive cells were calculated and compared between SE and SF culture conditions in DPSCs and SHED groups, independently. n = 3 for each group. Scale bar = 200 μm. SE, serum; SF, serum-free. Data shown as mean ± SEM. *p < 0.05. ns, not significant. **Fig. S3** Surface phenotypic profiles and in vitro immunoregulation ability of hMSC-DP. **a-b** Flow cytometry showed the percentage of CD146-positive hMSC-DP under SE or SF culture conditions at P5, P10 and P20 **(a)**. The percentage of CD146-positive cells was compared between SE and SF culture conditions **(b)**. n = 3 for each group. **c** The percentage of apoptotic T cells was calculated in SE and SF culture conditions. n = 3 for each group. SE, serum; SF, serum-free. Data shown as mean ± SEM. *p < 0.05. ns, not significant. **Fig. S4** Multilineage differentiation of hMSC-DP. **a** Alizarin red staining assay. The osteogenic capacity was compared between SE and SF culture conditions at P5, P10 and P20. n = 3 for each group. **b** Oil red O staining assay. The adipogenic capacity of hMSC-DP was compared between SE and SF culture conditions at P5, P10 and P20. n = 3 for each group. Scale bar = 50 μm. SE, serum; SF, serum-free. Data shown as mean ± SEM. **p < 0.01. ns, not significant. **Fig. S5** Therapeutic effects of hMSC-DP on experimental colitis. **a** Disease activity index (DAI) of DPSC- and SHED-treated groups was compared between SE and SF culture conditions at P5, P10 and P20. n = 3 for each group. **b** The colon length after DPSCs and SHED treatment was compared between SE and SF culture conditions at P5, P10 and P20. n = 3 for each group. **c** Histological structure was examined by H&E staining. The histological score of each group was compared between SE and SF culture conditions. n = 3 for each group. Scale bar = 100 μm. SE, serum; SF, serum-free. Data shown as mean ± SEM. ns, not significant. **Fig. S6** The expression level of CD146 is associated with hMSC-DP properties. **a** The correlation of CD146 with experimental parameters. **b** Linear regression plots showed the correlation between CD146 expression and experimental parameters including CFU-F clones, percentage of EdU positive cells, percentage of Alizarin red positive area, percentage of SA-β-gal positive cells, DAI scores, HAI scores and colon length.

**Additional file 2**. **Table S1**. Summary of clinical trials of hMSC-DP.

## Data Availability

All data generated or analyzed during this study are included in this published article and its Additional files.

## Abbreviations

hMSC-DP: Human mesenchymal stem cells from dental pulp
DPSCs: Postnatal human dental pulp stem cells
SHED: Stem cells from human exfoliated deciduous teeth
SE: Serum
SF: Serum-free
MSCs: Mesenchymal stem cells
ISCT: International Society for Cellular Therapy
SOPs: Standard operation procedures
FBS: Fetal bovine serum
EdU: 5-Ethynyl-20-deoxyuridine
PD: Population doublings
CFU: Colony-forming unit
WGA: Wheat germ agglutinin
β-gal: β-Galactosidase
SA-β-gal: Senescence-associated β-galactosidase
Runx2: Runt-related transcription factor 2
ALP: Alkaline phosphatase
PPARγ: Peroxisome proliferator-activated receptor-γ2
LPL: Lipoprotein lipase
DSS: Dextran Sulfate Sodium
DAI: Disease activity index
HAI: Histological activity index
