# Supplementary material for: CD146 controls the quality of clinical grade mesenchymal stem cells from human dental pulp
Source: Stem Cell Res Ther. 2021 Aug 30;12:488. doi: 10.1186/s13287-021-02559-4 (PMC8404346; doi:10.1186/s13287-021-02559-4)
Supplement: Supplementary file 1 — Additional file 1: Fig. S1 Characterization of the morphology of hMSC-DP under SF culture conditions. a-b Cell morphology analysis by high-content imaging. The nuclear area (a) and cell area (b) of DPSCs and SHED are shown in SE and SF culture conditions. n = 3 for each group. c Cellular senescence assay. The percentage of SA-β-gal positive cells was calculated and compared between SE and SF culture conditions. n = 3 ~ 5 for each group. SE, serum; SF, serum-free. Data shown as mean ± SEM. ns, not significant. Fig. S2 Proliferation capacity of hMSC-DP under SF culture conditions. a CFU-F assay. The numbers of colonies of hMSC-DP under SE or SF culture conditions were calculated and compared. n = 3 for each group. b Population doubling scores were calculated and compared between SE and SF culture conditions in DPSCs and SHED groups, independently. n = 3 for each group. c EdU assay. The percentage of EdU-positive cells were calculated and compared between SE and SF culture conditions in DPSCs and SHED groups, independently. n = 3 for each group. Scale bar = 200 μm. SE, serum; SF, serum-free. Data shown as mean ± SEM. *p < 0.05. ns, not significant. Fig. S3 Surface phenotypic profiles and in vitro immunoregulation ability of hMSC-DP. a-b Flow cytometry showed the percentage of CD146-positive hMSC-DP under SE or SF culture conditions at P5, P10 and P20 (a). The percentage of CD146-positive cells was compared between SE and SF culture conditions (b). n = 3 for each group. c The percentage of apoptotic T cells was calculated in SE and SF culture conditions. n = 3 for each group. SE, serum; SF, serum-free. Data shown as mean ± SEM. *p < 0.05. ns, not significant. Fig. S4 Multilineage differentiation of hMSC-DP. a Alizarin red staining assay. The osteogenic capacity was compared between SE and SF culture conditions at P5, P10 and P20. n = 3 for each group. b Oil red O staining assay. The adipogenic capacity of hMSC-DP was compared between SE and SF culture conditions at P5, [file 13287_2021_2559_MOESM1_ESM.pdf]

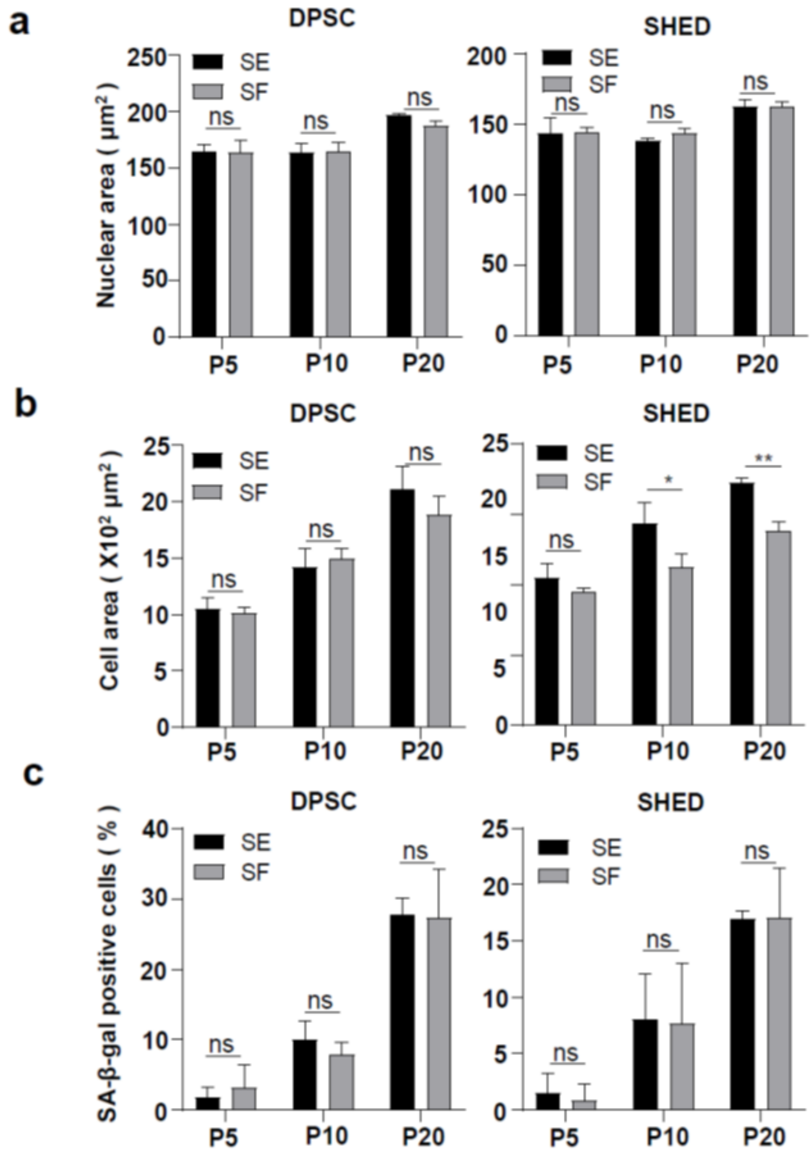

Fig. S1 Characterization of the morphology of hMSC-DP under SF culture conditions. **a-b** Cell morphology analysis by high-content imaging. The nuclear area **(a)** and cell area **(b)** of DPSCs and SHED are shown in SE and SF culture conditions. n = 3 for each group. **c** Cellular senescence assay. The percentage of SA-β-gal positive cells was calculated and compared between SE and SF culture conditions. n = 3 ~ 5 for each group. SE, serum; SF, serum-free. Data shown as mean ± SEM. ns, not significant.

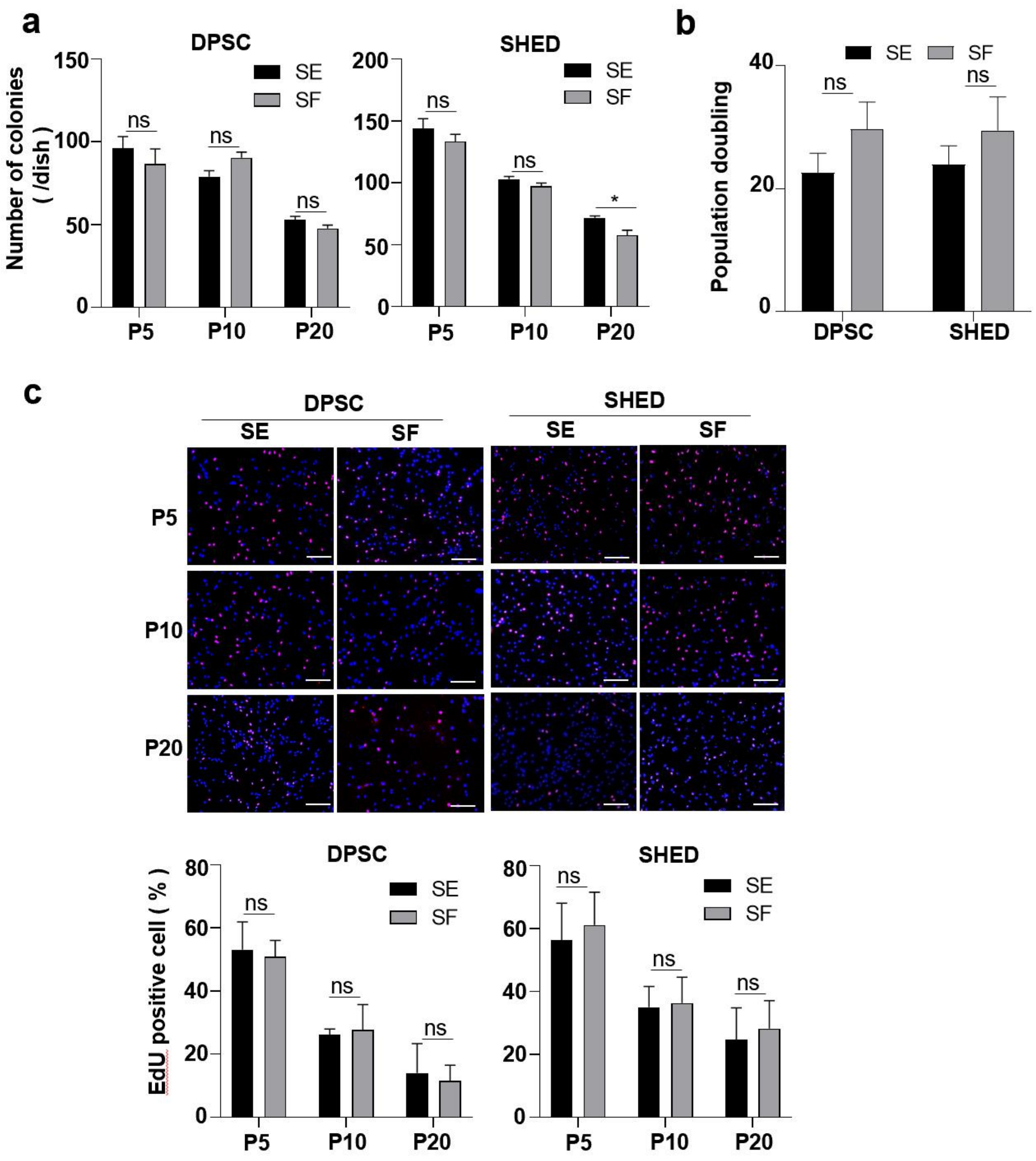

Fig. S2 Proliferation capacity of hMSC-DP under SF culture conditions. **a** CFU-F assay. The numbers of colonies of hMSC-DP under SE or SF culture conditions were calculated and compared. n = 3 for each group. **b** Population doubling scores were calculated and compared between SE and SF culture conditions in DPSC and SHED groups, independently. n = 3 for each group. **c** EdU assay. The percentage of EdU-positive cells were calculated and compared between SE and SF culture conditions in DPSC and SHED groups,

independently. n = 3 for each group. Scale bar = 200 μm. SE, serum; SF, serum-free. Data shown as mean ± SEM. *$p < 0.05$. ns, not significant.

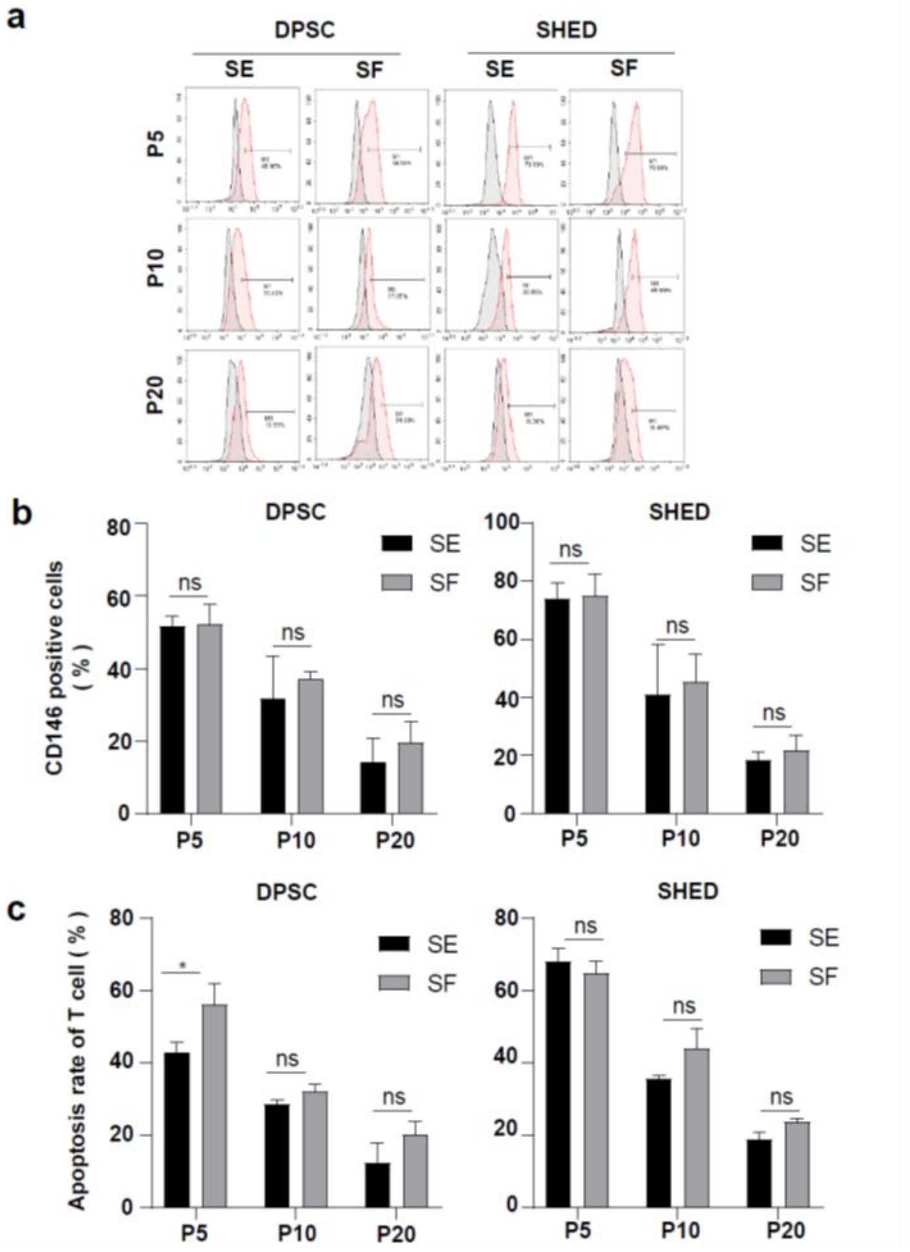

Fig. S3 Surface phenotypic profiles and *in vitro* immunoregulation ability of hMSC-DP. **a-b** Flow cytometry showed the percentage of CD146-positive hMSC-DP under SE or SF culture conditions at P5, P10 and P20 **(a)**. The percentage of CD146-positive cells was compared between SE and SF culture conditions **(b)**. n = 3 for each group. **c** The percentage of apoptotic T cells was calculated in SE and SF culture conditions. n = 3 for each group. SE, serum; SF, serum-free. Data shown as mean ± SEM. *$p < 0.05$. ns, not significant.

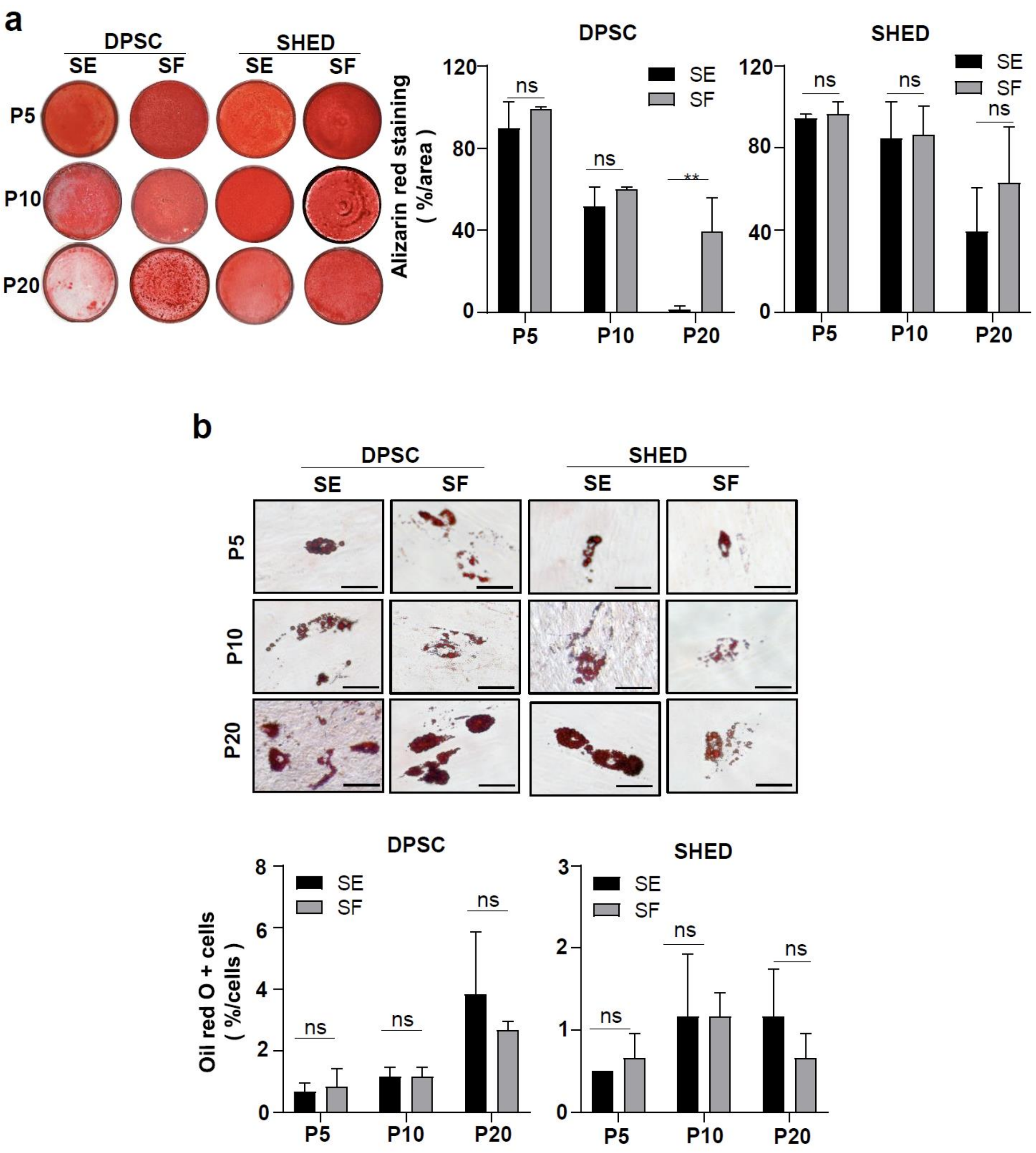

Fig. S4 Multilineage differentiation of hMSC-DP. **a** Alizarin red staining assay. The osteogenic capacity was compared between SE and SF culture conditions at P5, P10 and P20. n = 3 for each group. **b** Oil red O staining assay. The adipogenic capacity of hMSC-DP was compared between SE and SF culture conditions at P5, P10 and P20. n = 3 for each group. Scale bar = 50 μm. SE, serum; SF, serum-free. Data shown as mean ± SEM. $^{**}p < 0.01$. ns, not significant.

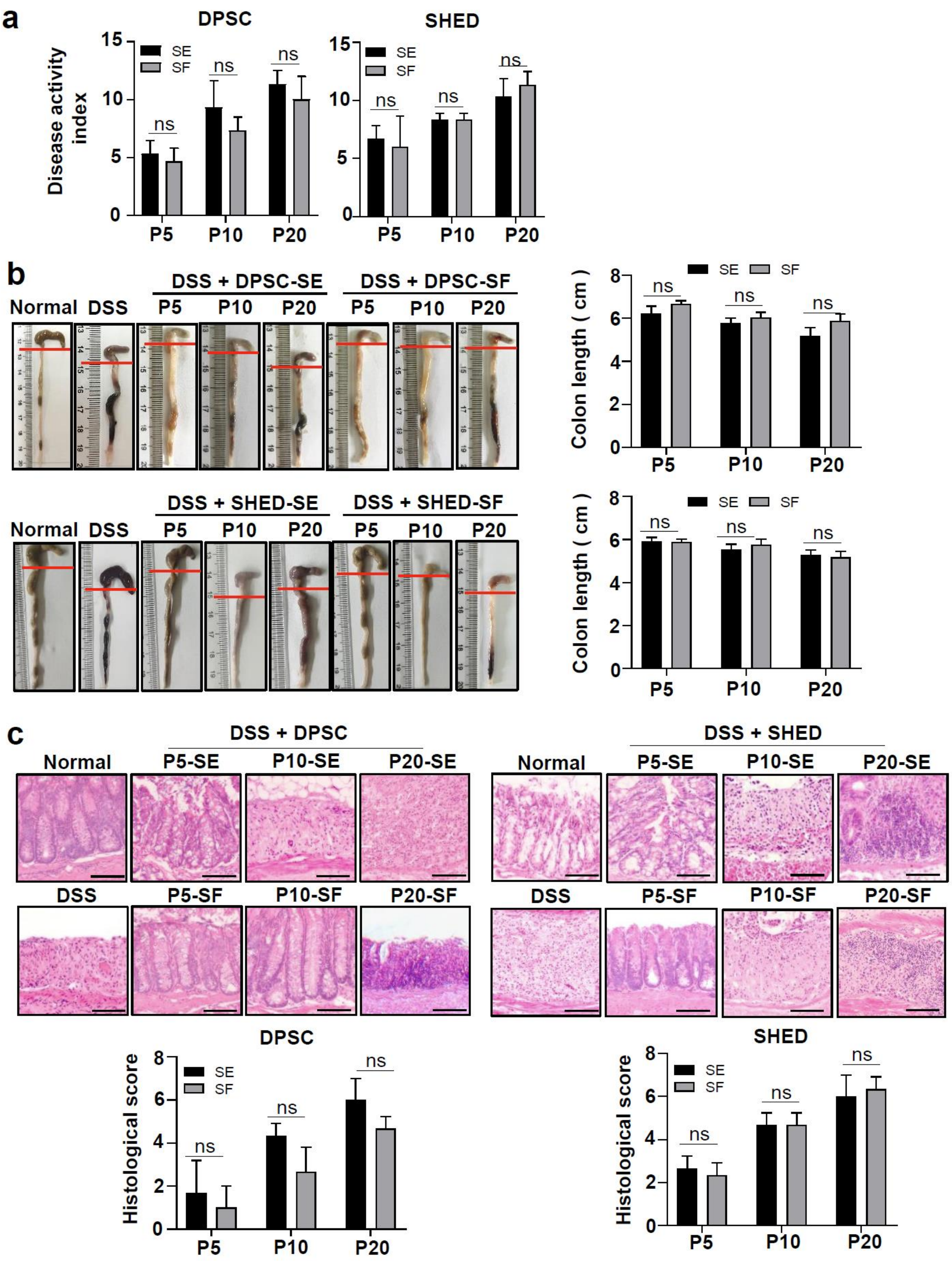

Fig. S5 Therapeutic effects of hMSC-DP on experimental colitis. **a** Disease activity index (DAI) of DPSC- and SHED-treated groups was compared between SE and SF culture conditions at P5, P10 and P20. n = 3 for each group. **b** The colon length after DPSC and SHED treatment was compared between SE and SF culture conditions at P5, P10 and P20. n = 3 for each group. **c**

Histological structure was examined by H&E staining. The histological score of each group was compared between SE and SF culture conditions. n = 3 for each group. Scale bar = 100 μm. SE, serum; SF, serum-free. T Data shown as mean ± SEM. ns, not significant.

a

hMSC-DP<br>R square<br>items	DPSC	SHED
CFU-F clones	0.8162	0.9416
EdU positive cells (%)	0.9429	0.8791
SA-β-gal positive cells (%)	0.9338	0.9237
Alizarin red staining (%/area)	0.9365	0.7800
Apoptosis rate of T cell (%)	0.9231	0.9888
DAI scores of DSS-induced colitis	0.9645	0.7568
HAI scores of DSS-induced colitis	0.9447	0.9846
Colon length (cm)	0.7787	0.8851

b

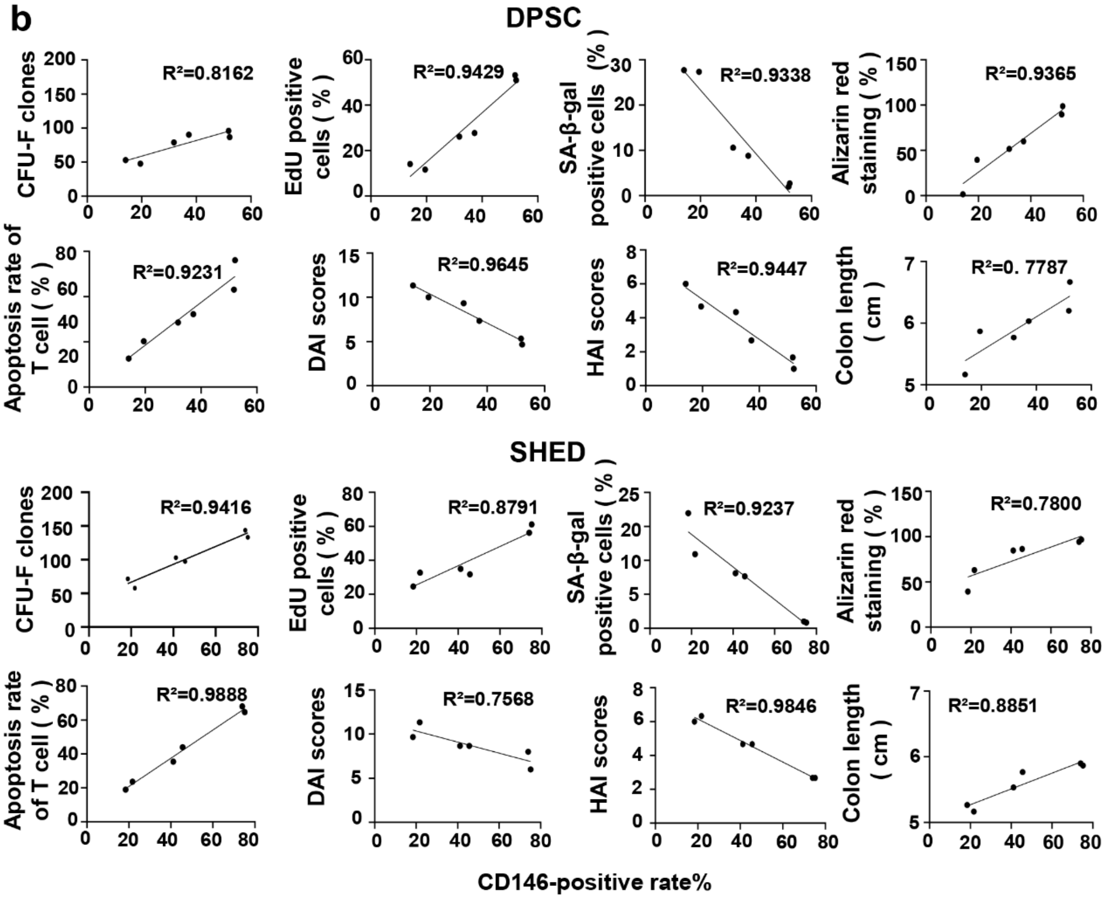

Fig. S6 The expression level of CD146 is associated with hMSC-DP properties. **a** The correlation of CD146 with experimental parameters. **b** Linear regression plots showed the correlation between CD146 expression and experimental parameters including CFU-F clones, percentage of EdU positive cells, percentage of Alizarin red positive area, percentage of SA-β-gal positive cells, DAI scores, HAI scores and colon length.
