# Supplementary material for: CD146 controls the quality of clinical grade mesenchymal stem cells from human dental pulp
Source: Stem Cell Res Ther. 2021 Aug 30;12:488. doi: 10.1186/s13287-021-02559-4 (PMC8404346; doi:10.1186/s13287-021-02559-4)
Supplement: Supplementary file 2 — Additional file 2. Table S1. Summary of clinical trials of hMSC-DP. [file 13287_2021_2559_MOESM2_ESM.docx]

|  | **NCT Number** | **Title** | **Status** | **Conditions** | **Locations** |
| --- | --- | --- | --- | --- | --- |
| **1** | NCT01814436 | Revitalization of Immature Permanent Teeth With Necrotic Pulps Using SHED Cells (Tissue regeneration) | Unknown | Dental Pulp Necrosis  Permanent Incisor Avulsed by Trauma | School of Stomatology, Fourth Military Medical University  Xi'an, Shaanxi, China |
| **2** | NCT01932164 | Use of Mesenchymal Stem Cells for Alveolar Bone Tissue Engineering for Cleft Lip and Palate Patients (Tissue regeneration) | Completed | Cleft Lip and Palate | Hospital Sírio Libanês  São Paulo, Brazil |
| **3** | NCT02523651 | Periodontal Regeneration of Chronic Periodontal Disease Patients Receiving Stem Cells Injection Therapy (Tissue regeneration) | Unknown | Periodontal Diseases | Capital Medical University School of Stomatology Beijing, Beijing, China |
| **4** | NCT02731586 | Effect on Allogenic Mesenchymal Stem Cells on Osseointegration of Dental Implants | Unknown | Edentulous Alveolar Ridge | Sri Sai College Of Dental Surgery and Research Centre  Vikarabad, Telangana State, India |
| **5** | NCT02842515 | Feasibility of the Preparation of an Advanced Therapy Medicinal Product for Dental Pulp Regeneration (Tissue regeneration) | Completed | Dental Stem Cells  Dental Pulp Regeneration |  |
| **6** | NCT03386877 | Periodontal Regeneration Using Dental Pulp Stem Cells (DPSCs) (Tissue regeneration) | Completed | Periodontal Diseases | CIR dental school Torino, Piedmont, Italy  CIR Dental school Turin University  Turin, Piedmont, Italy |
| **7** | NCT03766217 | Bone Tissue Engineering With Dental Pulp Stem Cells for Alveolar Cleft Repair (Tissue regeneration) | Completed | Cleft Lip and Palate | Hospital Sírio-Libanes São Paulo, Brazil |
| **8** | NCT03912480 | Stem Cells From Human Exfoliated Teeth in Treatment of Diabetic Patients With Significantly Reduced Islet Function | Unknown | Type 1 diabetes | Changhai hospital Shanghai, Shanghai, China |
| **9** | NCT03957655 | Safety and Efficacy of SHED for Decompensated Liver Cirrhosis | Not yet recruiting | Liver Cirrhosis | Changhai Hospital Shanghai, Shanghai, China |
| **10** | NCT04130100 | Clinical Study of Pulp Mesenchymal Stem Cells in the Treatment of Primary Mild to Moderate Knee Osteoarthritis (Immune therapy) | Recruiting | Knee Osteoarthritis | Changhai hospital  Shanghai, China |
| **11** | NCT04302519 | Novel Coronavirus Induced Severe Pneumonia Treated by Dental Pulp Mesenchymal Stem Cells (Immune therapy) | Not yet recruiting | COVID-19 |  |
| **12** | NCT04336254 | Safety and Efficacy Study of Allogeneic Human Dental Pulp Mesenchymal Stem Cells to Treat Severe COVID-19 Patients (Immune therapy) | Recruiting | COVID-19 | Renmin Hospital of Wuhan University (East Campus) Wuhan, Hubei, China |
| **13** | NCT04608838 | A Randomized Placebo-controlled Multicenter Trial to Evaluate the Efficacy and Safety of JTR-161, Allogeneic Human Dental Pulp Stem Cell, in Patients With Acute Ischemic stRoke (J-REPAIR) (Immune therapy) | Active, not recruiting | Acute Ischemic Stroke | Nippon Medical School Hospital  Bunkyo-ku, Tokyo, Japan |
| **14** | NCT04641533 | Effect of Dental Pulp Stem Cells and L-PRF After Impacted Third Molar Extraction | Completed | Stem Cell  Third Molar  Periodontal Pocket | Baskent University  Ankara, Turkey |

**Supplementary Table 1. Summary of clinical trials of hMSC-DP**
